# Supplementary material for: Identification of Wheat Ideotype under Multiple Abiotic Stresses and Complex Environmental Interplays by Multivariate Analysis Techniques
Source: Plants (Basel). 2023 Oct 11;12(20):3540. doi: 10.3390/plants12203540 (PMC10610392; doi:10.3390/plants12203540)
Supplement: Supplementary file 1 [file plants-12-03540-s001.zip › plants-2634488-supplementary.pdf]

Table S1. Names and pedigree of the 20 bread wheat genotypes (6 cultivars and 14 doubled haploid lines (DHLs)) used in this study.

Name	Pedigree
Gemmeiza-9	Ald"s"/Huac//CMH74 .630/SxCGM 4583 -5GM- 1GM- OGM
Gemmeiza-12	OTUS/3/SARA/THB//VEEMSS97Y00227S-5y-010M-010Y-010M-2Y-1M-0Y-OGM
Sakha-93	Sakha 92/TR810328 S8871-IS-2S-IS-0S
Misr1	OASSIS / SKAUZ//4*BCN/3/2*PATOR CMSS00Y01881T-050M-030Y-030M-030WGY-33M-0Y-0S
Pavone-76	Vcm//Cno/7C/3/Kal/Bb
KSU106	Barouk/R1474-75-3-53-3-3
DHLs (23,25)	Derived from the cross (Line-115 × Gemmeiza-7) (El-Hennawy et al. 2011)
DHLs (5,7,8,11)	Derived from the cross (Line-115 × Giza-164) (El-Hennawy et al. 2011)
DHLs (12,14,15,26,29)	Derived from the cross (Gemmeiza-7× Giza-164) (El-Hennawy et al. 2011)
DHLs (1,2, 6)	Derived from the cross (Giza-164× Giza-168) (El-Hennawy et al. 2011)

Table S2. Details of genotype codes and names of 20 bread wheat genotypes (6 cultivars and 14 doubled haploid lines (DHLs)) used in this study

Genotypes	Genotype code	Genotypes	Genotype code
DHL12	G1	DHL14	G11
DHL02	G2	DHL29	G12
DHL25	G3	DHL15	G13
DHL07	G4	DHL06	G14
DHL26	G5	Misr1	G15
Gemmeiza-9	G6	DHL05	G16
DHL11	G7	DHL23	G17
KSU106	G8	Sakha-93	G18
Gemmeiza-12	G9	Pavone-76	G19
DHL01	G10	DHL08	G20

Table S3. Monthly agro-climatological data at the experimental location during the growing seasons.

Parameters / Months	Precipitation (mm)			Temperature (°C)									Relative Humidity (%)		
				Maximum			Minimum			Average					
	S1	S2	S3	S1	S2	S3	S1	S2	S3	S1	S2	S3	S1	S2	S3
November	0.31	0.27	0.17	35.37	34.82	28.07	5.05	5.84	16.37	20.21	20.33	22.03	41.69	41.21	41.34
December	0.02	0.10	0.05	27.40	27.46	21.84	4.01	4.95	10.58	15.71	16.21	16.10	45.31	44.36	55.45
January	0.10	0.05	0.10	29.34	28.65	19.87	1.23	2.01	8.32	15.29	15.33	14.06	40.19	40.68	47.37
February	0.00	0.00	0.00	33.79	32.43	24.66	1.21	2.08	11.62	17.50	17.26	18.19	28.44	29.26	32.89
March	0.00	0.00	0.00	35.80	36.19	28.65	6.98	7.01	15.77	21.39	21.60	22.41	25.69	26.02	28.79
April	0.98	1.02	0.98	39.91	38.63	33.70	14.13	15.11	21.53	27.02	26.87	27.73	31.50	30.24	31.38
May	0.01	0.00	0.01	42.91	41.77	38.97	19.21	19.94	24.87	31.06	30.86	32.37	17.69	17.05	19.69

S1, Season 2018/2019; S2, Season 2019/2020, S3, Season 2020/2021.
